# Usefulness of DXA-based bone strain index in postmenopausal women with type 2 diabetes mellitus

**DOI:** 10.1007/s11657-024-01411-5

**Published:** 2024-06-26

**Authors:** Gloria Bonaccorsi, Guido Sciavicco, Luca Rinaudo, Andrea Brigato, Giuliana Fiorella, Aldo Carnevale, Fabio Massimo Ulivieri, Carmelo Messina

**Affiliations:** 1 Department of Translational Medicine, Menopause and Osteoporosis Center, University of Ferrara, Ferrara, Italy; 2 Department of Mathematics and Computer Science, University of Ferrara, Ferrara, Italy; 3 Tecnologie Avanzate T.A. S.R.L, Lungo Dora Voghera 36/A, Turin, Italy; 4 Department of Translational Medicine - Section of Radiology, University of Ferrara, Ferrara, Italy; 5 Bone Metabolic Unit, Casa Di Cura La Madonnina, Milan, Italy; 6 U.O.C. Radiodiagnostica, ASST Centro Specialistico Ortopedico Traumatologico Gaetano Pini-CTO, 20122 Milan, Italy; 7 Dipartimento Di Scienze Biomediche Per La Salute, Università Degli Studi Di Milano, Via Pascal, 36, Milan, Italy

**Keywords:** Osteoporosis, Type 2 diabetes, Bone strain index, BSI, Trabecular bone score, Menopause

## Abstract

***Summary*:**

Bone Strain Index (BSI) is a new dual-energy x-ray absorptiometry (DXA)-based index. We retrospectively evaluated data from 153 postmenopausal women with a history of type 2 diabetes mellitus (T2DM). Lumbar spine and femoral Bone Strain Index (BSI) were sensitive to skeletal impairment in postmenopausal women suffering from T2DM.

**Purpose:**

Bone Strain Index (BSI) is a new dual-energy X-ray absorptiometry (DXA)-based measurement. We evaluated the performance of BSI in predicting the presence of fragility fractures in type 2 diabetes mellitus (T2DM) postmenopausal women.

**Methods:**

We retrospectively evaluated data from a case–control study of 153 postmenopausal women with a history of at least 5 years of T2DM (age from 40 to 90 years). For each subject, we assessed the personal or familiar history of previous fragility fractures and menopause age, and we collected data about bone mineral density (BMD), BSI, and Trabecular Bone Score (TBS) measurements. Statistical analysis was performed having as outcome the history of fragility fractures.

**Results:**

Out of a total of 153 subjects, *n* = 22 (14.4%) presented at least one major fragility fracture. A negative correlation was found between lumbar BSI and lumbar BMD (*r* =  − 0.49, *p* < 0.001) and between total femur BSI and total femur BMD (*r* =  − 0.49, *p* < 0.001). A negative correlation was found between femoral neck BSI and femoral neck BMD (*r* =  − 0.22, *p* < 0.001). Most DXA-based variables were individually able to discriminate between fractured and non-fractured subjects (*p* < 0.05), and lumbar BSI was the index with the most relative difference between the two populations, followed by femoral BSI.

**Conclusion:**

Lumbar spine and femoral BSI are sensitive to skeletal impairment in postmenopausal women suffering from T2DM. The use of BSI in conjunction with BMD and TBS can improve fracture risk assessment.

## Introduction

Menopause and diabetes are two well-known risk factors for osteoporosis, with diabetes being more frequent with increasing age and often coexisting with osteoporosis [1]. Subjects with type 2 diabetes mellitus (T2DM) are at increased fracture risk [2, 3] despite the paradoxical behavior of areal bone mineral density (BMD) values measured by dual-energy X-ray absorptiometry (DXA), which often show to be higher in T2DM individuals compared to non-diabetic ones [4, 5]. Despite DXA remains the reference technique for bone status assessment and osteoporosis diagnosis [6], it is also known that this technique suffers from limitations, mainly because low BMD accounts for about 70% of the fragility fractures [7, 8]. This is true for T2DM subjects and can be explained by the fact that other factors play a role in influencing bone strength and fracture risk, such as bone microarchitecture and bone deformation capability when a load is applied [9, 10].

To overcome this limitation, several DXA-based tools have been developed over time, such as the Trabecular Bone Score (TBS), an indirect DXA bone microarchitecture index used since 2008 [4]. TBS showed to be able to discriminate fractured patients independently from BMD and predict fragility fractures both in primary and secondary osteoporosis, including T2DM [11–13]. However, TBS does not provide data about femoral bone quality status and does not consider all the necessary information to evaluate the performance of bone under loads [14].

Recently, a new index has been developed using a different mathematical approach, the finite element analysis (FEA). This index evaluates the DXA distribution of density measured on both spine and femoral scans and has been developed with the name Bone Strain Index (BSI). BSI is inferred from lumbar (L-BSI) and femur (F-BSI) DXA scans, and its calculation considers information on density distribution, bone geometry, weight, and the specific load applied on the anatomic site. It diverges from BMD and TBS, as it is based on the quantification of bone mass and its distribution over the scanned area. In addition to bone density and its distribution, BSI includes data concerning the shape of the skeletal investigated site and in-specific conditions load applied to the bone by means of the patient’s weight. BSI calculation, in fact, is based on a mathematical approach (FEA) that consists of simplifying a complex object into simpler elements to which the laws of classical mechanics apply. Forces and constraints applied to the bone in specific areas generate internal stresses and strains, which depend on the magnitude and the type of solicitation, the bone geometry, and the stiffness of each simple element in which the bone has been divided [8, 14].

In primary osteoporosis, L-BSI and F-BSI were demonstrated to be able to predict all fragility fractures [15]. L-BSI is useful for the identification of osteoporotic female subgroups with particular tendency to first fragility fractures [16] and to successive fractures [17, 18]. Furthermore, low values of F-BSI are connected with the absence of vertebral fractures [19]. In secondary osteoporosis, L-BSI demonstrated good ability in discriminating vertebral fractured patients affected by hyperparathyroidism [20, 21] and appeared to be useful in the clinical characterization of patients affected by mastocytosis [22] and recessive dystrophic epidermolysis bullosa [23], whereas F-BSI proved to be associated with vertebral fractures in aromatase inhibitor-naive patients [24].

Several studies evaluated the ability of TBS to discriminate T2DM-fractured subjects from control subjects, showing that TBS values are lower in diabetic patients and that TBS is associated with fracture risk [4, 11, 12]. To our knowledge, no data are available about the capability of BSI to discriminate patients affected by diabetes and to predict fragility fractures. Therefore, our aim is to evaluate the performance of BSI in predicting the presence of fragility fractures in T2DM postmenopausal women.

## Methods

### Patient population

This is a case–control study performed in the Research Center for the Study of Menopause and Osteoporosis (CMO) at the University of Ferrara. From 2018 to 2022, a total of 153 subjects with T2DM were enrolled. Inclusion criteria were women aged between 40 and 90 years, postmenopausal status in accordance with the STRAW staging system [25], a history of at least 5 years of T2DM (all subjects being treated with oral hypoglycemic drugs or insulin), and normal kidney function. At the enrollment, none of them presented with patent evidence of microvascular complications such as retinopathy, nephropathy, or peripheral neuropathies. Exclusion criteria were the presence of medical disorders or treatments interfering with bone metabolism (such as bisphosphonates, teriparatide, denosumab, and corticosteroids) in the previous 2 years. As the study focused on a well-defined and homogeneous population (T2DM postmenopausal women), the variability was minimized rendering a large sample size unnecessary; additionally, as the research question was aimed at exploring qualitative aspects rather than quantifying effects, a formal sample size calculation was not strictly necessary.

For each subject, we collected data to assess the history of previous major fragility fractures at hip or lumbar spine and the menopause age. The evaluation of glucose metabolism was done with routine laboratory assay for each subject by measuring fasting glycemia and glycated hemoglobin (HbA1c) levels. Data were collected from the CMO digital archive of those women attending the center for performing a DXA scan for routine BMD assessment. The study was approved by local Ethical Committee, and each subject signed anonymized data authorization for scientific publication.

The densitometer installed at CMO is connected to a Windows-based computer with a patented software that can manage DXA exam with archiving and recall functions. For each DXA scan, a file in PDF format is produced containing all relevant information (bone mineral content and BMD, *T*-score, and *Z*-score values) including those risk factors that are relevant for FRAX™ score calculation. Every scan generates a new record, which is archived in a MySQL database and is integrated with different fields (or attributes) that can be filled by the physician, such as clinical history, previous treatments, current treatments, current diagnosis, and follow-up recommendations.

### BMD, BSI, and TBS measurements

The evaluation of densitometric parameters was performed using DXA (Hologic Discovery; software version APEX 3.3.0.1, Bedford MA, USA) to assess BMD values at both lumbar spine and proximal femur. DXA examinations were performed according to manufacturer’s manual and the International Society for Clinical Densitometry (ISCD) official positions [26]. Regarding the lumbar spine, we included the L1–L4 region whenever possible, with fractured or degenerative sclerotic vertebrae being excluded from the analysis when a difference of more than 1.0 T-score value was found. At least two vertebrae were used for the analysis. Regarding the proximal femur, BMD analysis was conducted at femoral neck and total femur regions.

TBS values of lumbar spine were obtained from the same region used for BMD analysis, using the TBS Insight software (Medimaps, version 2.1). We excluded from the TBS analysis subject with BMI below 15 kg/m^2^ and above 37 kg/m^2^, according to manufacturer software limitations [4].

BSI values were from DXA raw using the BSI software version 1.4.0 (Tecnologie Avanzate s.r.l., Torino, Italy) which is installed on the same DXA computer. This software extrapolates BSI values at the same region of interest of BMD at lumbar spine and proximal femur (L1–L4, femoral neck, and total femur). The final BSI value accounts for the average bone equivalent strain of the specific region, assuming that the higher BSI, the higher the strain level, as well as the risk of fracture. On the contrary, lower BSI values indicate a bone subjected to lower strain and lower fracture risk [14]. The reported in vivo coefficient of variation (CV) for the BSI is 4.17% and 3.89% for femoral neck and total femur, respectively, and 1.79% for the lumbar spine [27, 28]. For the abovementioned studies, the BSI CV was associated with a corresponding BMD CV, which is similar to those of our center. Therefore, we can assume that our CV for the BSI is comparable to those reported in recent literature.

### Statistical analysis

The analysis was performed having as outcome the history of major fragility fractures at spine, proximal femur, proximal humerus, and distal radius. The normality of distribution of variables was assessed using the Shapiro–Wilk test. For those variables that did not behave normally, data are reported as median and interquartile range (IQR). The correlation between variables of the two groups (non-fractured vs. fractured subjects) was done with the Pearson test or Kendall test depending on the normality status of each variable, defined as follows: < 0.10, negligible; 0.10–0.40, weak; 0.41–0.69, moderate; 0.71–0.89, strong; and 0.90–1.00, very strong [27]. The predictive power of each single variable towards the presence of a fragility fracture was assessed by performing a univariate analysis (Student *t*-test/Mann–Whitney test, depending on the normality status of each variable). A *p* value lower than 0.05 was considered significant in all tests, although it must be taken into consideration that the population counts less than 500 subjects. Receiver operating characteristic (ROC) curves were estimated to predict the possible use of DXA-based variables in distinguishing fractured and non-fractured subjects. Finally, we performed a multivariate analysis with the intent of establishing the predictive power of sub-groups of variables towards the presence of a fragility fracture in a subject; we operated in leave-one-out cross-validation mode to compensate for the low cardinality of the dataset. We also considered several combinations of variables to establish the amount of predictive power that BSI to predict a fragility fracture, by testing several intelligent knowledge extraction techniques.

To evaluate BSI predictive ability, alone and in combination with BMD and TBS, we applied simple learning techniques to study the behavior of the models as the parameters vary. Our approach is based on using several different classifiers with the aim of establishing the predictive power in a model-independent way; more specifically, we applied linear discriminant analysis, logistic regression, support vector machine, and random forests. In all cases, we defined the problem as classification one and in leave-one-out cross-validation mode on maximal stratified subsets (40 repetitions). We explored combinations using TBS, BMD, BSI only, TBS + BMD, TBS + BSI, BMD + BSI, and TBS + BMD + BSI.

## Results

Out of a total of 153 subjects, *n* = 131 (85.6%) did not present a fragility fracture, while *n* = 22 (14.4%) presented at least one major fragility fracture. More in detail, eighteen subjects presented only vertebral fractures, one subject presented only femoral fractures, and three presented both vertebral and femoral fractures. A total of *n* = 36 (23%) presented other fragility fractures, including wrist, proximal humerus, and ribs. The descriptive analysis of the overall population is presented in Table 1.

**Table 1:** Descriptive analysis of the study population according to the different variables

Indices	Minimum	Maximum	Mean	Stand. Dev	Diff. mean	Median	IQR	Normality
Age (years)	40	87	66.6	9.4	0.1	67	13.5	1
Age at menopause (years)	27	59	49.3	5.1	0.04	50	5	0
Glycated HB (mmol/mol)	33	106	51.64	11.24	0.03	50	12	0
Glycemia (mg/dL)	59	290	123.72	38.272	0.049	113	38	0
BMI (kg/m^2^)	15.9	36.9	27.8	4.8	0.02	27.7	7.2	1
FRAX (major fracture)	1.6	46.0	9.6	7.8	1.8	6.9	7.5	0
FRAX (femoral neck)	0.1	28.0	3.2	4.6	2.7	1.5	3.0	0
TBS corrected FRAX (Major fracture)	1.4	44	9.8	7.9	1.8	7.3	8.4	0
TBS corrected FRAX (Femoral neck)	0	26.3	3.3	4.7	3.2	1.5	3.6	0
TBS	0.810	1.489	1.242	0.122	0.074	1.248	0.146	1
Neck BMD (g/cm^2^)	0.381	1.066	0.693	0.127	0.169	0.674	0.168	0
Femoral BMD (g/cm^2^)	0.500	1.255	0.863	0.146	0.147	0.873	0.172	1
Lumbar BMD (g/cm^2^)	0.600	1.368	0.935	0.158	0.065	0.92	0.202	0
Femoral BSI	0.91	2.62	1.56	0.30	0.21	1.52	0.28	0
Neck BSI	0.94	3.19	1.86	0.43	0.20	1.83	0.47	0
Lumbar BSI	0.91	3.98	2.05	0.56	0.16	2.04	0.68	0

*HB*, hemoglobin; *BMI*, body mass index; *TBS*, Trabecular Bone Score; *BMD*, bone mineral density; *BSI*, Bone Strain Index. Normality = 1 indicates that the population behaves normally for that variable

After the initial descriptive assessment, we applied a simple outlier elimination strategy. The inclusion criteria were menopause age > 35, glycemia < 250 mg/dL, glycated HB < 90, and null values in variables of the third group < 10%. After the elimination of outliers, the dataset contained overall 137 subjects, 116 (84.7%) non-fractured subjects, and 21 (15.3%) fractured subjects.

Pearson and Kendall correlation analyses were performed, and results are shown in Table 2. A negative correlation was found between lumbar BSI and lumbar BMD (*r* =  − 0.49, *p* < 0.001) and between total femur BSI and total femur BMD (*r* =  − 0.49, *p* < 0.001). A negative correlation was found between femoral neck BSI and femoral neck BMD (*r* =  − 0.22, *p* < 0.001). A positive correlation was found between TBS and lumbar BMD (*r* = 0.29, *p* < 0.001).

**Table 2:**
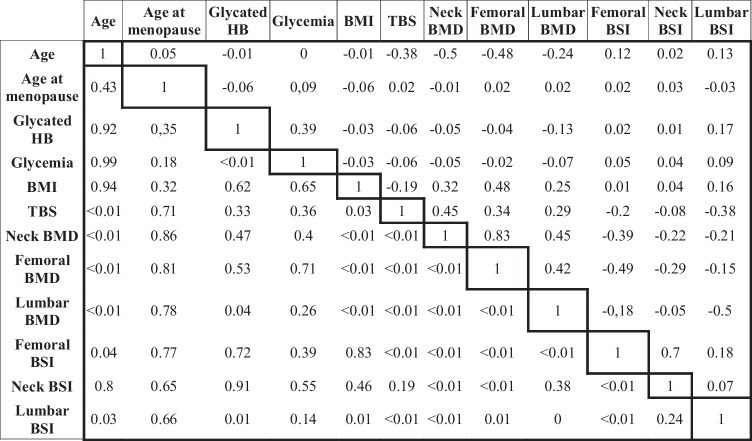
Pearson and Kendall correlation analysis. The upper-right part shows correlation indices, while the lower-left part the matching *p* values

*HB*, hemoglobin; *BMI*, body mass index; *TBS*, Trabecular Bone Score; *BMD*, bone mineral density; *BSI*, Bone Strain Index

**Fig. 1:**
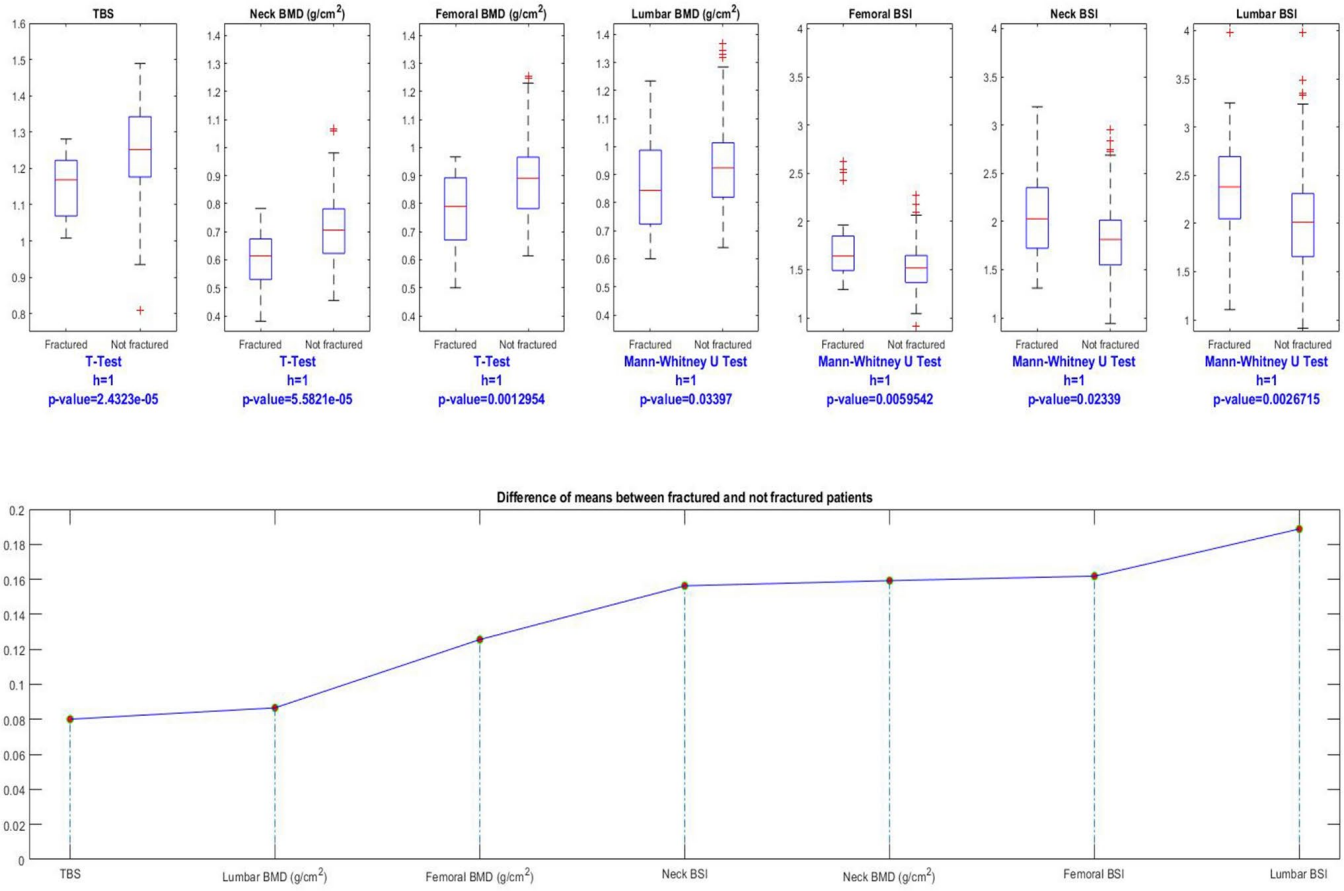
Boxplot showing the differences in the distribution of the different analyzed variables across the two subgroups. For each variable, the statistical test we used and the *p* value are reported at the bottom. HB, hemoglobin; BMI, body mass index; TBS, Trabecular Bone Score; BMD, bone mineral density; BSI, Bone Strain Index

**Fig. 2:**
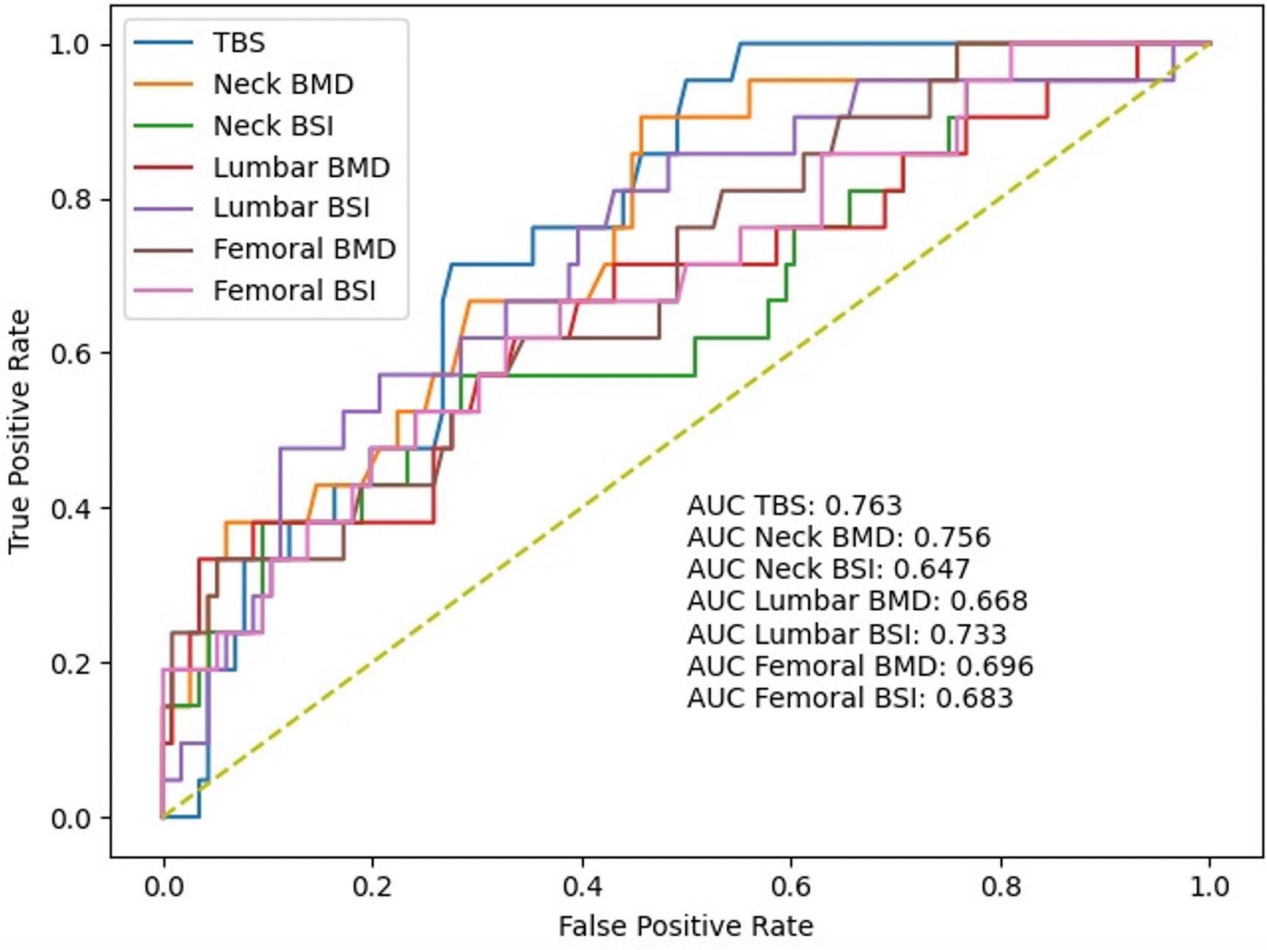
Receiver operating characteristic (ROC) curves evaluating the discriminatory power of the different DXA-based variables

Univariate analysis showed that most of the considered variables were individually able to discriminate between fractured and non-fractured subjects. Subjects with major fragility fractures presented with significantly lower values (*p* < 0.05) of femoral BMD (both neck and total), lumbar BMD and TBS, and BSI parameter (lumbar, neck, and total femur). Boxplots showing the clear difference in the distribution of variables among the two subgroups are reported in Fig. 1. When comparing the differences, lumbar BSI emerges as the index with the most relative difference between the two populations, followed by femoral BSI. This can be appreciated at the bottom of Fig. 1, which shows the comparison between normalized mean of values among the several indexes and their difference between the two groups: as it can be seen, the most significant one emerged for lumbar BSI. ROC curve analysis to distinguishing fractured and non-fractured subjects revealed that the optimal AUC was reached by TBS (0.763) followed by femoral neck BMD (0.756) and lumbar BSI (0.733). The overall diagnostic performances of the different variables are reported in Fig. 2.

Results from multivariate analysis are reported in Table 3. As it can be seen, the average values that emerge in terms of accuracy, sensitivity, and specificity of classification when one type of index is employed (that is, TBS, BMD, or BSI only) are the lowest ones except for BSI specificity. The values tend to grow when the classifiers are given more elaborate combinations of indexes, and the greatest ones emerge when all DXA-related indexes are used. While absolute values seem not very high, they are very reliable. Indeed, the differences between the combinations have been tested as populations, and the corresponding *p* values show what follows. The BSI specificity is greater than either BMD (*p* value <  < 0.01) and TBS (*p* value 0.003) and not lesser when indexes are combined. In particular, BSI specificity is greater than specificity of BMD + TBS combination (*p* value 0.0004) and not different for the other ones. Furthermore, when either TBS or BMD or both are added to the BSI, the accuracy and sensitivity but not the specificity of learned model increases. When the BSI is added to BMD or TBS all performance increases.

**Table 3:** Multivariate analysis. Each column respectively displays the average value of accuracy, sensitivity, and specificity of the average of 40 executions of each classifier (linear discriminant analysis, logistic regression, support vector machine, and random forests); each execution corresponds to a different balanced downsampling

Index combinations	Accuracy	Sensitivity	Specificity
TBS	0.62 ± 0.1	0.6 ± 0.12	0.64 ± 0.1
BMD all sites	0.59 ± 0.08	0.59 ± 0.1	0.6 ± 0.08
BSI all sites	0.65 ± 0.07	0.61 ± 0.07	0.7 ± 0.09
BMD all sites + BSI all sites	0.68 ± 0.07	0.68 ± 0.09	0.68 ± 0.09
BMD all sites + TBS	0.66 ± 0.08	0.68 ± 0.1	0.64 ± 0.08
TBS + BSI all sites	0.69 ± 0.07	0.68 ± 0.08	0.7 ± 0.09
TBS + BMD all sites + BSI all sites	0.69 ± 0.07	0.69 ± 0.08	0.7 ± 0.08

## Discussion

In this study we explored, for the first time, the role of BSI in evaluating fracture risk among postmenopausal women with type 2 diabetes (T2DM), comparing it with BMD and TBS. Our primary findings indicate that BSI, as a novel DXA-derived index, proves to be valuable in distinguishing between T2DM postmenopausal women with and without fragility fractures.

Over the years, several papers were aimed at evaluating the role of BMD and DXA-based parameters to assessing T2DM skeletal status and the related risk of fragility fractures. In fact, it is well-known that the skeleton is affected by T2DM, increasing the risk of fragility fractures of these individuals [28]. Nevertheless, the role of BMD by DXA has been questioned over time, because it is generally higher in T2DM subjects compared to non-diabetics [11]. As a result, additional parameters have been assessed under the assumption that the reduction in bone strength is not solely linked to BMD alone.

Regarding TBS, robust evidence shows that this DXA-based parameter index is lower in T2DM than non-diabetics, differently than BMD [11, 29]. The first large study was conducted by Leslie et al. in 2013, who retrospectively studied 2356 diabetic women from the Manitoba cohort; lower values of TBS were reported in the diabetic cohort, despite higher BMD values at both spine and femur compared to non-diabetic subjects [11]. In 2016, Bonaccorsi et al. confirmed the role of TBS as a tool to increase the diagnostic accuracy to discriminate fragility fracture in the context of T2DM secondary osteoporosis [12]. In the evaluation of fracture risk, it has been demonstrated that the performance of TBS surpasses that of BMD in predicting fractures among individuals with diabetes. Consequently, the practice of combining BMD and TBS is deemed effective in enhancing the accuracy of fracture risk prediction [13].

Our study showed that DXA-based variables could discriminate between fractured and non-fractured subjects, including BSI. When comparing the differences between groups, we demonstrated that lumbar BSI and femoral BSI are the two indices with the higher relative difference between groups, suggesting the capability of BSI in predicting the presence of fragility fractures in T2DM subjects. The valuable role of BSI emerged also from the multivariate analysis, as the accuracy of the learned model for classifying a subject with or without a fragility fracture increased substantially when BSI was combined to other variables as BMD or TBS. Of note, the accuracy values we found are obtained in leave-one-out cross-validation mode, which in the case of small datasets—such as ours—guarantees the most reliable results. This explains why our values are not too high in absolute terms.

Correlation analysis showed that only weak or moderate correlation was found between BSI and BMD. On the one hand, the significance of these correlations is related to the fact that the BSI calculation takes into account BMD and is influenced by its values. Nevertheless, BSI evaluates different aspects compared to BMD: it includes not only information related to BMD but also from specific patient loading and bone geometry characteristic but also bone density distribution [14]. Therefore, this correlation may suggest that BSI is able to provide information on fracture risk that is somewhat independent of BMD.

Our study has limitations, firstly related to its retrospective nature. Another limitation is that we included a relatively small number of subjects with fragility fractures. Finally, being a single-center study, the analysis was done on a single densitometer; therefore, the applicability of our results to other machines and manufacturers remains to be assessed.

BSI offers a different insight on bone resistance to fracture, which is different from TBS and BMD. In fact, BSI uses the finite element analysis to simulate gravitational force at each bony segment, ultimately assessing the strain distribution. TBS is an indirect index of microarchitecture, while BMD accounts basically for the bone quantity [14]. Hip geometry evaluates the macroarchitecture of proximal femur starting from a two-dimensional DXA scan, to extrapolate geometrical parameters associated with fracture risk [30]. Of note, BSI takes into account information from hip geometry to obtain the final stress value. Currently, existing BSI thresholds indicate poor resistance when BSI is equal or lower than 2.5 [31]. Such cut-off values can be applied also to enhance the fracture risk assessment in conditions like T2DM.

In conclusion, lumbar spine and femoral BSI are sensitive to skeletal impairment in postmenopausal women suffering from T2DM. The use of BSI in conjunction with BMD and TBS improved the fracture risk assessment, showing its usefulness in the setting of secondary osteoporosis due to diabetes. In addition, the use of BSI in combination with other DXA-base indices may result in better therapeutic appropriateness. Further work is needed in this field to better understand the effect of disorders like T2DM on BSI and its related mechanism, as well as for BSI in other secondary causes of osteoporosis.

## Data Availability

All data are available on request to the corresponding author.
